# *Eutremananum* (Brassicaceae), a new species from Chola Shan, Southwest China

**DOI:** 10.3897/phytokeys.109.27049

**Published:** 2018-09-17

**Authors:** Guoqian Hao, Ihsan A. Al-Shehbaz, Lei Zhang, Xinyi Guo, Hao Bi, Songbai Xu, Jianquan Liu

**Affiliations:** 1 Biodiversity Institute of Mount Emei, Mount Emei Scenic Area Management Committee, Leshan 614000, Sichuan, P. R. China; 2 Key Laboratory for Bio-Resources and Eco-Environment of Ministry of Education, College of Life Science, Sichuan University, Chengdu 610065, Sichuan, P. R. China; 3 Missouri Botanical Garden, P.O. Box 299, St. Louis, MO 63166-0299, USA

**Keywords:** Cruciferae, *
Eutrema nanum
*, molecular phylogeny, Sichuan, *
Eutrema sinense
*

## Abstract

*Eutremananum*, a new high-elevation (4500–4600 m) species from Chola Shan, Sichuan (Southwest China), is described and illustrated. It is similar morphologically to *E.nepalense* but is readily distinguished by having oblong to elliptic or obovate to spatulate (vs. suborbicular to broadly ovate) leaves, glabrous (vs. puberulent) sepals and ovate to oblong fruit 4–7 × 2–3 mm with flattened valves (vs. ovoid to subglobose fruit 2–3 × 1.8–2 mm with rounded valves). The genetic differences amongst *E.nanum*, *E.nepalense* and other close relatives are further confirmed by phylogenetic analyses using ITS and cpDNA sequence variations. The new combination *E.sinense* is proposed.

## Introduction

The boundaries of *Eutrema* R.Br. (Brassicaceae or Cruciferae) have recently been expanded to include 38 species, several of which were previously placed in the six genera *Taphrospermum* C.A.Mey., *Thellungiella* O.E.Schulz, *Neomartinella* Pilg., *Platycraspedum* O.E.Schulz, *Chalcanthus* Boiss. and *Pegaeophyton* Hayek & Hand.-Mazz. (Al-Shehbaz and Warwick 2005; Hao et al. 2017a). The taxonomic knowledge of this genus is still incomplete because numerous collections from the high-elevation regions in Southwest China were often overlooked and many areas remain poorly explored. We reported two new species during recent field investigations and molecular analyses (Hao et al. 2016, 2017b). Here we report the third one, *Eutremananum*, found in Chola Shan at a high elevation of 4500–4600 m in Sichuan Province, Southwest China. This new species is morphlogically similar to *E.nepalense* (Al-Shehbaz, Kats Arai & H.Ohba) Al-Shehbaz, G.Q.Hao & J.Quan Liu but, as shown below, it is readily distinguished by several aspects of leaves and fruit. The phylogenetic studies on both species and their other relatives were also conducted herein and the results support the recognition of this novelty. In addition, one of six species which were used to determine the systematic position of *E.nanum* was found to need a taxonomic combination and a new name *Eutremasinense* (Hemsl.) G.Q.Hao, J.Quan Liu & Al-Shehbaz is therefore proposed herein.

## Material and methods

We examined morphological traits of *Eutremananum* and several relative species. We followed Hu et al. (2015) and Hao et al. (2017a) in examining the genetic differences between this novelty (two accessions) and the morphologically similar *E.nepalense* (one accession). In order to determine the systematic position of *E.nanum*, we futher included six species (*E.scapiflorum* (Hook.f. & Thomson) Al-Shehbaz, G.Q.Hao & J.Quan Liu, *E.sinense* (Hemsl.) G.Q.Hao, J.Quan Liu & Al-Shehbaz, *E.hookeri* Al-Shehbaz & Warwick, *E.fontanum* (Maxim.) Al-Shehbaz & Warwick, *E.verticillatum* (Jeffrey & W.W.Sm.) Al-Shehbaz & Warwick and *E.deltoideum* (Hook.f. & Thomson) O.E.Schulz) in our analyses. All six species were shown to be close relatives to *E.nepalense* in our previous study (Hao et al. 2017a) and two (*E.scapiflorum* and *E.sinense*) were previously placed in the genus *Pegaeophyton*. The related *E.integrifolium* Bunge (see Hao et al. 2017a) was selected as the outgroup. The collection information of the sampled species is listed in Table 1 and Figure 3 and the voucher specimens were deposited in the Sichuan University Herbarium (SZ).

**Table 1.** The sources of materials used for molecular analyses of Himalayan *Eutrema* (all vouchers at SZ).

Taxon	Voucher	Source	Coordinate
* E. nanum *	Liu & Hao 14091	Chola Shan, Sichuan, China	31°55'N, 98°54'E
* E. nanum *	Liu 17124	Chola Shan, Sichuan, China	31°55'N, 98°54E
* E. nepalense *	Long et al. 605	Sikkim, India	27°36'N, 88°12'E
* E. sinense *	Liu 13114	Biluo Snow Mountain, Yunnan, China	27°59'N, 98°47'E
* E. scapiflorum *	Liu & Hao 13074	Yarla Shampo Mountain, Tibet, China	28°51'N, 91°59E
* E. fontanum *	Liu & Hao 13144	Zhuodala Mountain, Sichuan, China	31°24'N, 99°56'E
* E. hookeri *	Liu 17108a	Mila Mountain, Tibet, China	29°49'N, 92°90'E
* E. verticillatum *	Liu & Hao 14094	Maila Mountain, Sichuan, China	30°58'N, 98°58'E
* E. deltoideum *	Liu 13024	Lasa, Tibet, China	29°42'N, 91°09'E
* E. integrifolium *	Liu & Hao 13049	Tianshan Mountain, Xinjiang, China	43°12'N, 84°49'E

We extracted the total DNA and amplified and sequenced four DNA markers, the nuclear internal transcribed spacer (ITS) and three chloroplast DNA (cpDNA) regions (*psb*A-*trn*H, *rbc*L, *mat*K), following Hu et al. (2015) and Hao et al. (2017a). The sequences firstly reported here were placed in GenBank under the accession numbers (MH702367, MH793597, MH793598, MH793599). We aligned all sequences using Clustal X (Thompson et al. 1997) and refined them manually. We concatenated three cpDNA sequences into a single matrix for Maximum Parsimony (MP) and Maximum Likelihood (ML) analyses. We coded indels using the simple code method by GapCoder (Young and Healy 2003). We constructed phylogenetic relationships based on two datasets (ITS and cpDNAs) using MP analyses by PAUP* 4.10b (Swofford 2003) and ML analyses using RAxML 7.2.6 (Stamatakis 2006). MP analyses employed a heuristic search with 10,000 replicates and TBR branch swapping and bootstrap values (Felsenstein 1985) were estimated with 1000 replicates and 100 random-addition-sequence replicates per bootstrap replicate. ML analyses were performed with raxmlHPC -f a -s sequence. phy -n boot2 -m GTRGAMMA -x 1234 -# 1000 -n outname. The most suitable GTRGAMMA models were used and bootstrap analyses were estimated with 1000 replicates.

## Taxonomy

### Eutrema nanum

Taxon classificationFungiBrassicalesBrassicaceae

G.Q.Hao, J.Quan Liu & Al-Shehbaz sp. nov.

urn:lsid:ipni.org:names:60477015-2

#### Type.

**Figure 1.**
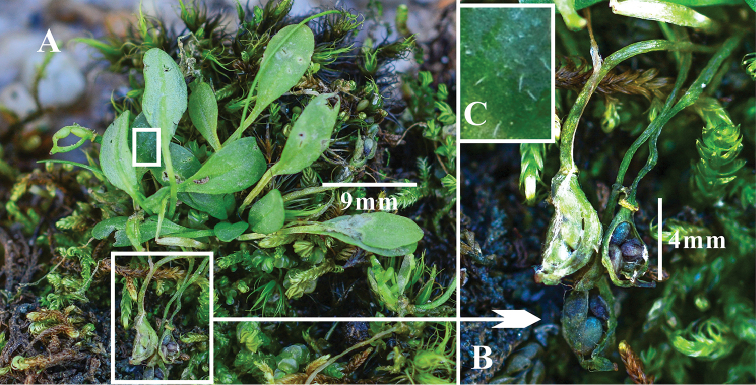
*Eutremananum.* G.Q. Hao, J.Quan. Liu & Al-Shehbaz. **A** Plant **B** Fruit **C** Leaf trichomes.

**Figure 2.**
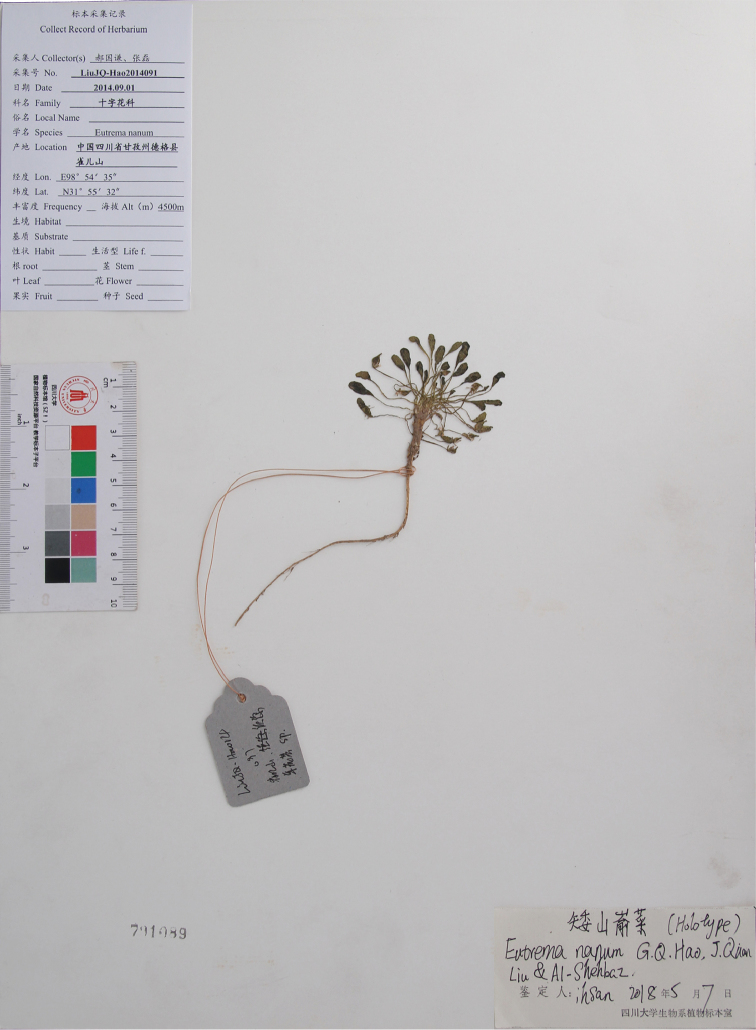
Holotype of *Eutremananum*.

China. Sichuan: Chola Shan, 31°55'32"N, 98°54'35"E, 4500 m elev., 16 August 2014, *Liu & Hao 14091* (Holotype, SZ). Figures 1, 2.

#### Description.

Herbs perennial, 3–6 cm tall, glabrous or puberulent; caudex slender, ca. 3–5 mm long. Leaves basal, rosulate, 20–25 per caudex; petiole 13–20 mm long, slender at base, glabrous or with few trichomes; blade oblong, elliptic, obovate, spatulate, 6–10 × 3–4 mm, fleshy, glabrous or abaxially pubescent with trichomes, 0.3–0.6 mm long, base subattenuate, to cuneate, margin entire, apex obtuse to subrounded. Pedicels slender, 18–23 mm long at anthesis, not elongated in fruit, not persistent. Flowers 5–8 per plant; sepals ovate to oblong, 1–1.5 mm long; petals white, broadly obovate to spatulate, blade 2–3 ×1–2 mm, persistent to fruit maturity, claw-like base 0.5–1 mm long. Ovules 2–4 per ovary. Fruit latiseptate, dehiscent, ovate to oblong, somewhat curved, 4–7 × 2–3 mm; valves nearly flat, extending along part of fruit length; gynophore 0.1–0.3 mm long; replum 0.3–0.4 mm wide; style 0.6–1 mm long. Seeds broadly ovate, brown, plump, 2–4 per fruit, 1.4–2 × 0.6–1 mm.

*Eutremananum* is morpholgically most similar to *E.nepalense*, from which it is readily distinguished by having oblong, elliptic, obovate to spatulate leaves, glabrous sepals and ovate to oblong larger fruit 4–7 × 2–3 mm with flattened, glabrous valves. In contrast, *E.nepalense* (https://www.gbif.org) has suborbicular to broadly ovate leaves, puberulent sepals and ovoid to subglobose smaller fruit 2–3 × 1.8–2 mm with rounded, puberulent valves. *Eutremananum* was only found with around 100 individuals along a stream in a valley about 2 kilometres from the Chola Shan peak, whereas *E.nepalense* occurs across Himalyas Mountains in Bhtan, China, Nepal and India.

#### Phenology.

Flowering: June–August. Fruiting: August–September.

#### Distribution and habitat.

**Figure 3.**
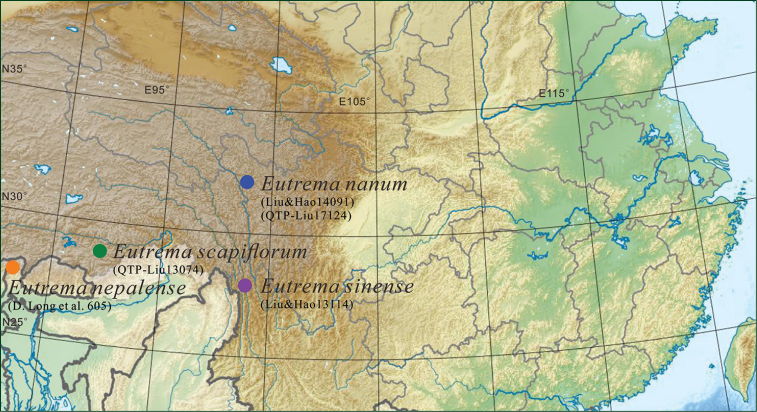
Geographical distribution of the sampled populations of *Eutremananum* and related species.

*Eutremananum* is currently known only from Chola Shan, part of Hengduan Mountains in West Sichuan, China (Fig. 3). It grows under rocks by streams close to glaciers, damp or gravelly scree, wet sand at a very high elevation of 4500–4600 m.

**Paratype**. China. Sichuan: Chola Shan, 31°55'32"N, 98°54'35"E, 4500 m elev., *Liu 17124* (SZ).

### Eutrema sinense

Taxon classificationFungiBrassicalesBrassicaceae

(Hemsl.) G.Q.Hao, J.Quan Liu & Al-Shehbaz, comb. nov. Based on Braya sinensis Hemsl., J. Linn. Soc., Bot. 30: 303. 1892.

urn:lsid:ipni.org:names:77190124-1

**Syn.**: Eutremarobustum (O.E.Schulz) Al-Shehbaz, G.Q.Hao & J.Quan Liu, Bot. J. Linn. Soc. 184: 2019. 2017. Basionym: Pegaeophytonsinensevar.robustum O.E.Schulz, Notizbl. Bot. Gart. Gerlin-Dahlem 9: 477. 1926.

The earliest available epithet of this taxon at the species rank is “*sinensis*” and it should have been been transferred to *Eutrema* by Hao et al. (2017a) instead of using the varietal epithet “*robustum*.”

### Genetic differences between *Eutremananum*, *E.nepalense* and other relatives

Sequence data from *Eutremananum* and *E.nepalense* reveals that one nucleotide substitution in ITS, two in *rbc*L, 18 in *mat*K and eight substitutions and three indels in *psb*A-*trn*H distinguish them very well (Table 2).

**Table 2.** Diagnosing sites of the aligned ITS and three cpDNA sequences between *Eutremananum* and *E.nepalense*.

Species	ITS	*rbc*L		*mat*K											
	508	82	337	165	276	333	342	391	449	483	495	497	549		
* Eutrema nanum *	C	C	T	C	T	T	T	T	T	T	C	T	T		
* Eutrema nepalense *	T	A	C	T	C	G	C	A	G	C	T	C	A		
	***mat*K**					***psb*A-*trn*H**									
	601	603	633	638	657	28	40	48	92	114	115	138-	212	228	235
* Eutrema nanum *	C	C	T	T	G	T	C	G	-	G	C	-	2 nt	C	C
* Eutrema nepalense *	T	T	C	A	A	A	G	A	6 nt	T	A	74 nt	-	T	A

nr=nucleotide.

**Figure 4.**
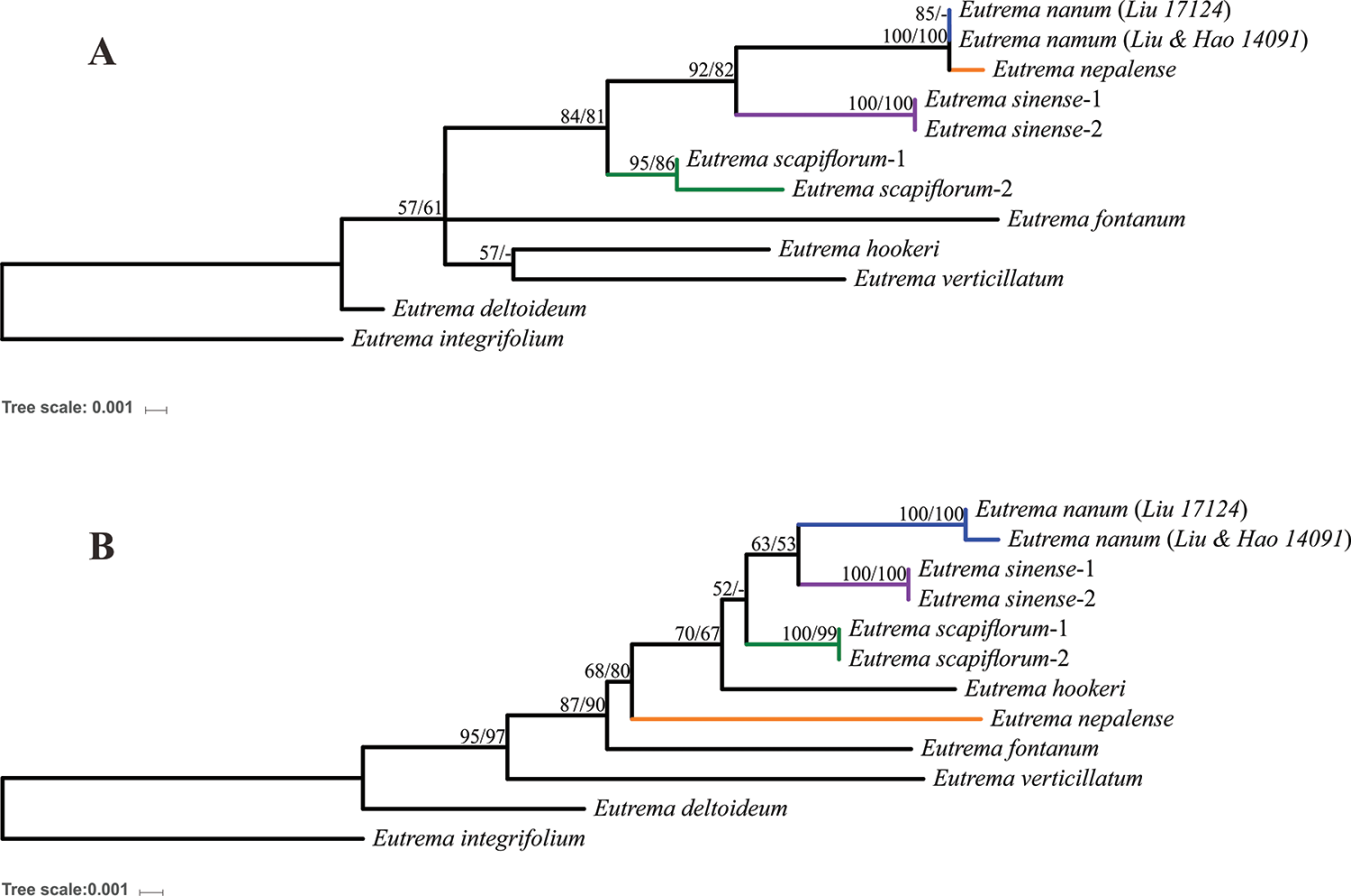
The Maximum Likelihood tree based on analysis of ITS (**A**) and Three cpDNA regions (**B**). Numbers above branches are maximum likelihood bootstrap support values and maximum parsimony bootstrap support values. ‘-’ represents <50%.

Based on sequence variations of ITS and cpDNAs (Table 3), phylogenetic analyses suggested that *Eutremananum* is mostly related to *E.nepalense*, *E.sinense* and *E.scapiflorum*. However, phylogenetic relationships of these four species are incongurent between ITS and plastid DNA tree (Fig. 4). In the ML analyses of ITS sequence data, *E.nanum* and *E.nepalense* formed a single cluster sister to *E.sinense* and together are sister to *E.scapiflorum* with high support values (>80%) (Fig. 4A). By contrast, in the ML analyses of cpDNAs sequences, the phylogenetic relationships were maintined between *E.nanum* and sister *E.sinense* and together as sister to *E.scapiflorum*, but *E.nepalense* fell outside that relationship and was separated from them by *E.hookeri* with medium support (>50%) (Fig. 4B). MP analyses produced almost the same tree topologies with similar bootstrap support values.

**Table 3.** Tree statistics for analyses of the datasets.

Data set	ITS^*^	*psb*A-*trn*H	*rbc*L	*mat*K	Combined cpDNA^*^
No. of sequences	30	30	30	30	30
Aligned length used in analyses	698	455	506	779	1786
No. of variable characters	141	58	17	66	169
No. of parsimony-informative characters	56	15	7	22	47
Tree length (steps)	78	70	10	71	195
Consistency (CI)	0.833333	0.900000	1.000000	0.873239	0.892308
Retention index (RI)	0.803030	0.708333	1.000000	0.790698	0.764045
Rescaled consistency index (RC)	0.669192	0.637500	1.000000	0.690468	0.681763

^*^ gaps were coded and included.

## Discussion

Both *Eutremananum* and *E.nepalense* are small plants similar in flower traits and seed size. However, as discussed above, they are quite different morphologically. In addtion, phylogenetic analyses of cpDNAs variations suggested these two species did not comprise a monophyletic clade. Furthermore, the Himalayan *E.nepalense* is disjunctly separated by a distance of at least 1200 air kilometres from the Chola Shan (Sichuan, SW China), where *E.nanum* is endemic (Fig. 3). *Eutremananum* is also closely related to *E.sinense* in the phylogenetic analyses of the cpDNA sequence variations, but both are easily distinguished from each other. *Eutremananum* is a small and weak herb with entire leaves (0.5–1.5 cm long) and small flowers (petals 2–3 mm long), whereas *E.sinense* is obviously stout with entire or toothed leaves (1.5–8 cm long) and distinctly larger flowers (petals 8–15 mm long) and fruit (10–20 mm long).

Pylogenetic relationships amongst *E.nanum*, *E.nepalense* and *E.sinense* are incongruent between ITS and cpDNA trees. This incongruence may suggest possible hybridisations or incomplete lineage sorting during the rapid and recent species diversifications (Soltis and Soltis 2000, 2009). However, it is not possible at present to determine which of these two factors had caused the incongruent phylogenies observed here. More analyses and molecular data, especially based on more individuals and genomic evidence, are needed to solve these phylogenetic inconsistences.

## Supplementary Material

XML Treatment for Eutrema nanum

XML Treatment for Eutrema sinense
